# TiO_x_N_y_ Modified TiO_2_ Powders Prepared by Plasma Enhanced Atomic Layer Deposition for Highly Visible Light Photocatalysis

**DOI:** 10.1038/s41598-018-30726-w

**Published:** 2018-08-14

**Authors:** Yan-Qiang Cao, Xi-Rui Zhao, Jun Chen, Wei Zhang, Min Li, Lin Zhu, Xue-Jin Zhang, Di Wu, Ai-Dong Li

**Affiliations:** National Laboratory of Solid State Microstructures and Department of Materials Science and Engineering, College of Engineering and Applied Sciences, Collaborative Innovation Center of Advanced Microstructures, Nanjing University, Nanjing, 210093 People’s Republic of China

## Abstract

In this work, TiN film deposited by plasma enhanced atomic layer deposition (PEALD) is adopted to modify the commercial anatase TiO_2_ powders. A series of analyses indicate that the surface modification of 20, 50 and 100 cycles of TiN by PEALD does not change the morphology, crystal size, lattice parameters, and surface area of TiO_2_ nano powders, but forms an ultrathin amorphous layer of nitrogen doped TiO_2_ (TiO_x_N_y_) on the powder surfaces. This ultrathin TiO_x_N_y_ can facilitate the absorption of TiO_2_ in visible light spectrum. As a result, TiO_x_N_y_ coated TiO_2_ powders exhibit excellent photocatalytic degradation towards methyl orange under the visible light with good photocatalytic stability compared to pristine TiO_2_ powders. TiO_x_N_y_ (100 cycles PEALD TiN) coated TiO_2_ powders exhibit the excellent photocatalytic activity with the degradation efficiency of 96.5% in 2 hours, much higher than that of pristine TiO_2_ powder of only 4.4%. These results clearly demonstrate that only an ultrathin surface modification layer can dramatically improve the visible light photocatalytic activity of commercial TiO_2_ powders. Therefore, this surface modification using ALD is an extremely promising route to prepare visible light active photocatalysts.

## Introduction

Titanium dioxide (TiO_2_) is the most widely investigated photocatalyst due to its good photocatalytic activity, high chemical and thermal stability, nontoxicity, low cost, and excellent degradation capacity^1–3^. However, a large band gap (3.2 eV) of TiO_2_ has limited its practical applications since it can be only activated by the illumination of ultra-violet light, which only makes up 4–5% of the solar spectrum^4^. In order to utilize a wider solar spectrum, it is highly desirable that the TiO_2_-based photocatalysts can work under visible light. Therefore, considerable efforts have been devoted for TiO_2_ to facilitate its visible light absorption. There are several ways can be applied to achieve this goal, such as element doping^5–7^ and coupling with metal or other semiconductors^8–11^. Among various approaches, non-metal doping of TiO_2_ has shown great promise in enhancing visible light active photocatalysis, with nitrogen doping being the most promising dopant^3,12,13^. N-doped TiO_2_ nanomaterials have been synthesized successfully by various methods, such as hydrolysis of TTIP in a water/amine mixture, post-treatment of the TiO_2_ sol with amines, ball milling of TiO_2_ in a NH_3_ water solution^14–16^. N-doped TiO_2_ nanomaterials could also be obtained by annealing TiO_2_ under NH_3_ flux at high temperature^17^. In addition, several film deposition techniques including sputtering^18^, chemical vapour deposition^19^, atomic layer deposition (ALD)^20^, have also been applied to prepare N-doped TiO_2_ film. The visible light photocatalytic activity of N-doped TiO_2_ nanomaterials has been explored thoroughly. Although the effect of N doping on photocatalytic enhancement of TiO_2_ is still debated, it is well accepted that N doping can cause the red shift absorption threshold of TiO_2_, improving the visible light photocatalytic activity^21,22^.

ALD is a novel and promising thin film deposition technique based on sequential self-limited and complementary surface chemisorption reactions, which is able to deposit ultrathin, uniform, and conformal layers, and it’s especially suitable for coating 3D complex structures. In recent years, ALD has attracted increasing attention in synthesis and surface engineering of complex nanostructures in recent years^23–26^. ALD has shown great prospects in various applications, such as lithium ion batteries^27,28^, supercapacitors^29–31^, catalysis^32,33^, and solar energy conversions^34^. Plasma enhanced ALD (PEALD), employing plasma as one precursor, has shown some merits over conventional thermal ALD (T-ALD), such as higher film density, lower impurity, higher growth rate, better electronic properties. Moreover, less energy is required to drive the surface reaction because of the high reactivity of plasma species, resulting in a lower deposition temperature^35^.

Various N-doped TiO_2_ nanomaterials, which exhibit highly visible light photocatalytic performance, have been successfully synthesized. However, the effect of ultrathin N-doped TiO_2_ surface coating/modification on visible light photocatalysis of TiO_2_ has not been well researched. Herein, PEALD was adopted to deposit ultrathin TiN film on TiO_2_ powders. The deposited TiN film would be oxidized into TiO_x_N_y_ (N doped TiO_2_) when exposed to the air, achieving TiO_x_N_y_ coated TiO_2_ after PEALD TiN coating. This ultrathin TiO_x_N_y_ coating can facilitate the visible light absorption of TiO_2_. Correspondingly, the TiO_x_N_y_ coated TiO_2_ powders exhibit significantly enhanced visible light photocatalytic activity towards methyl orange (MO) and phenol degradation.

## Results

Bui *et al*. have reported that the surface of deposited TiN film would be oxidized when exposed to the air^36^. Therefore, the XPS spectra of the TiN film deposited by PEALD on silicon were firstly conducted to explore the surface chemistry of as-deposited TiN, as shown in Fig. S1. Both Ti-O and Ti-N bonding can be detected in Ti 2*p* spectra, confirming the formation of TiO_x_N_y_ on the PEALD TiN surface, in consistent with reported literature^36^. Therefore, it can be speculated that TiO_x_N_y_ coated TiO_2_ composite can be achieved here after coating ultrathin PEALD TiN on TiO_2_ surface.

**Figure 1:**
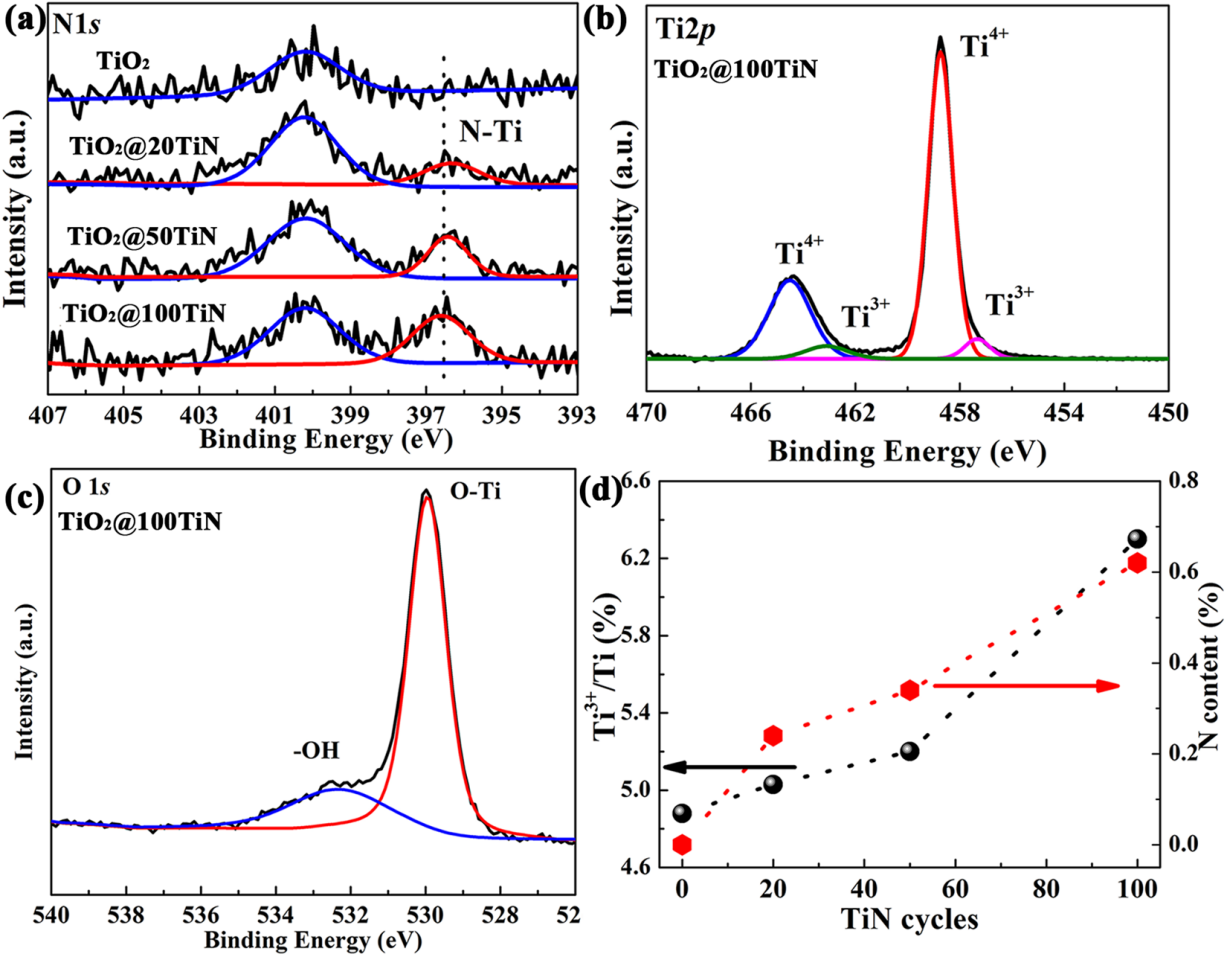
(**a**) N 1s XPS spectra of pristine TiO_2_ and TiO_x_N_y_ coated TiO_2_ powders, (**b**) Ti 2p and (**c**) O1s spectra of TiO_2_@100TiN, (**d**) Ti^3+^/Ti ratio and N content verse TiN coating cycles.

Next, the surface chemical nature of PEALD TiN coated TiO_2_ catalyst was also characterized by XPS. XPS spectra were fitted with Gaussian-Lorentzian (G-L) functions after smart-type background subtraction. Figure 1a shows the N 1 s spectra of pristine TiO_2_ and PEALD TiN coated TiO_2_. It can be found that pristine TiO_2_ only exhibits a weak peak at ~400.1 eV, which can be assigned to absorptive nitrogen molecules^37,38^. After PEALD TiN coating, there appears a new peak at 396.5 eV, corresponding to the formation of N-Ti bonding^37,38^. And the intensity of N-Ti is enhanced with increasing the PEALD TiN cycles. In Ti 2p spectra of pristine TiO_2_ (Fig. S2a), the doublet at 464.4 and 458.7 eV can be assigned to Ti 2p_1/2_ and Ti 2p_3/2_ peaks of Ti-O bonds with the spin orbit splitting energy of 5.7 eV, consistent with the value of TiO_2_^11^. Besides, there are two weak doublet peaks at 462.9 eV and 457.2 eV can also be detected, which can be assigned to Ti^3+^ defects on the surface^39^. More Ti^3+^ can be introduced onto the surface of TiO_2_ after PEALD TiN coating. Therefore, the surface Ti^3+^/Ti ratio increases with increasing PEALD deposition cycles, as shown in Fig. 1d. All the samples show the similar O 1s spectra with main O-Ti bonds of TiO_2_ at 529.9 eV, as shown in Fig. 1c and Fig. S2, the peak at 532.4 eV related to the -OH on the surface can also be detected^7^. Figure 1d illustrates the plots of surface Ti^3+^/Ti ratio and N content versus the PEALD TiN cycles, it can be seen that both surface Ti^3+^ and N content increase with PEALD TiN cycles. Combined with XPS data of PEALD TiN film on Si (Fig. S1), it can be concluded that TiO_x_N_y_ coating layer was formed on TiO_2_ surface after ultrathin PEALD TiN coating. It has been proved in previous literatures that both Ti^3+^ and N sites can narrow the band gap of TiO_2_^40,41^. Therefore, it can be speculated that a much smaller band gap can be achieved for the TiO_x_N_y_ layer, promoting the visible light absorption.

**Figure 2:**
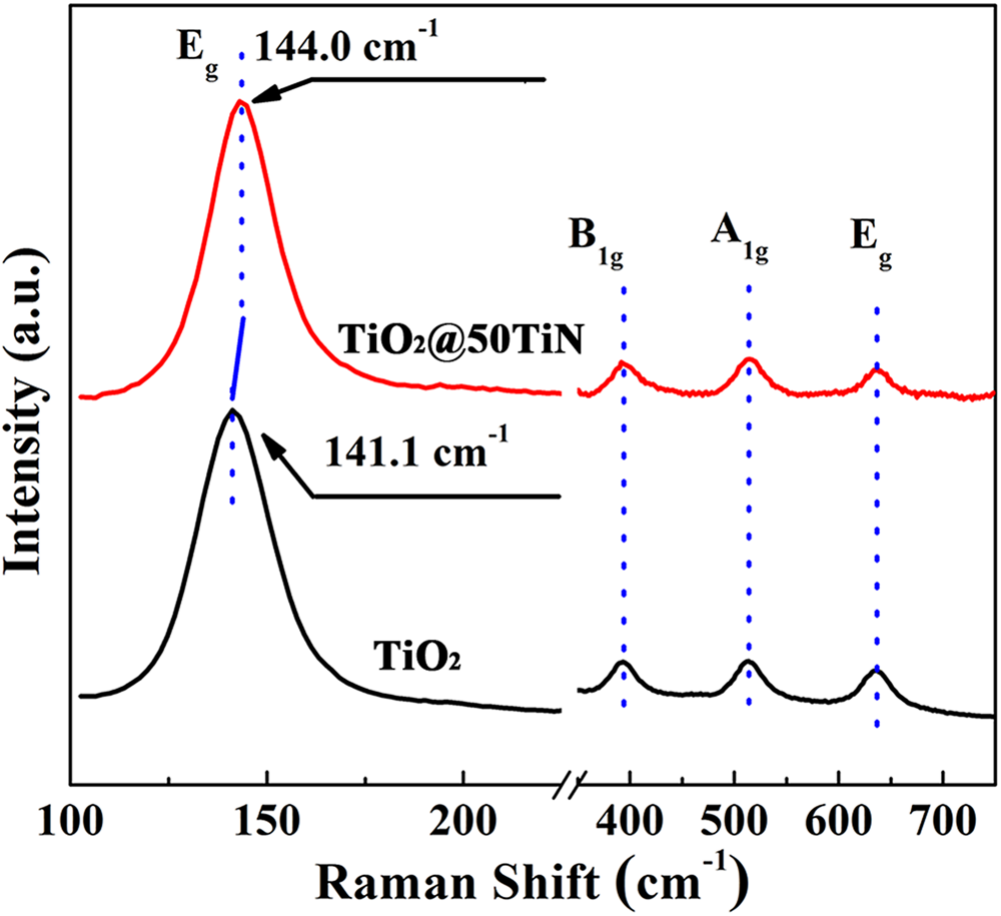
Raman spectra of pristine TiO_2_ and TiO_2_@50TiN powders.

Figure 2 shows the Raman spectra of pristine TiO_2_ and TiO_2_@50TiN prepared by PEALD. According to the previously reported data^42^, the anatase phase of TiO_2_ has six Raman bands at 144 cm^−1^ (Eg), 197 cm^−1^ (Eg), 399 cm^−1^ (B1g), 513 cm^−1^ (A1g), 519 cm^−1^ (B1g) and 639 cm^−1^ (Eg), and the rutile phase has four Raman bands at 143 cm^−1^ (B1g), 447 cm^−1^ (Eg), 612 cm^−1^ (A1g), and 826 cm^−1^ (B2g). Both samples here present Raman spectra the same as the pure anatase phase, with no peaks related to the rutile phase. More importantly, the most remarkable feature is that the predominant peak position (Eg) undergoes a blue shift from 141.1 cm^−1^ to 144.0 cm^−1^ after TiO_x_N_y_ modification. Previous literatures have demonstrated that the N doping in TiO_2_ can result in the blue shift for Eg mode^43^. Therefore, the slight blue shift here can be ascribed to the small amount TiO_x_N_y_ formation on the TiO_2_ surface.

**Figure 3:**
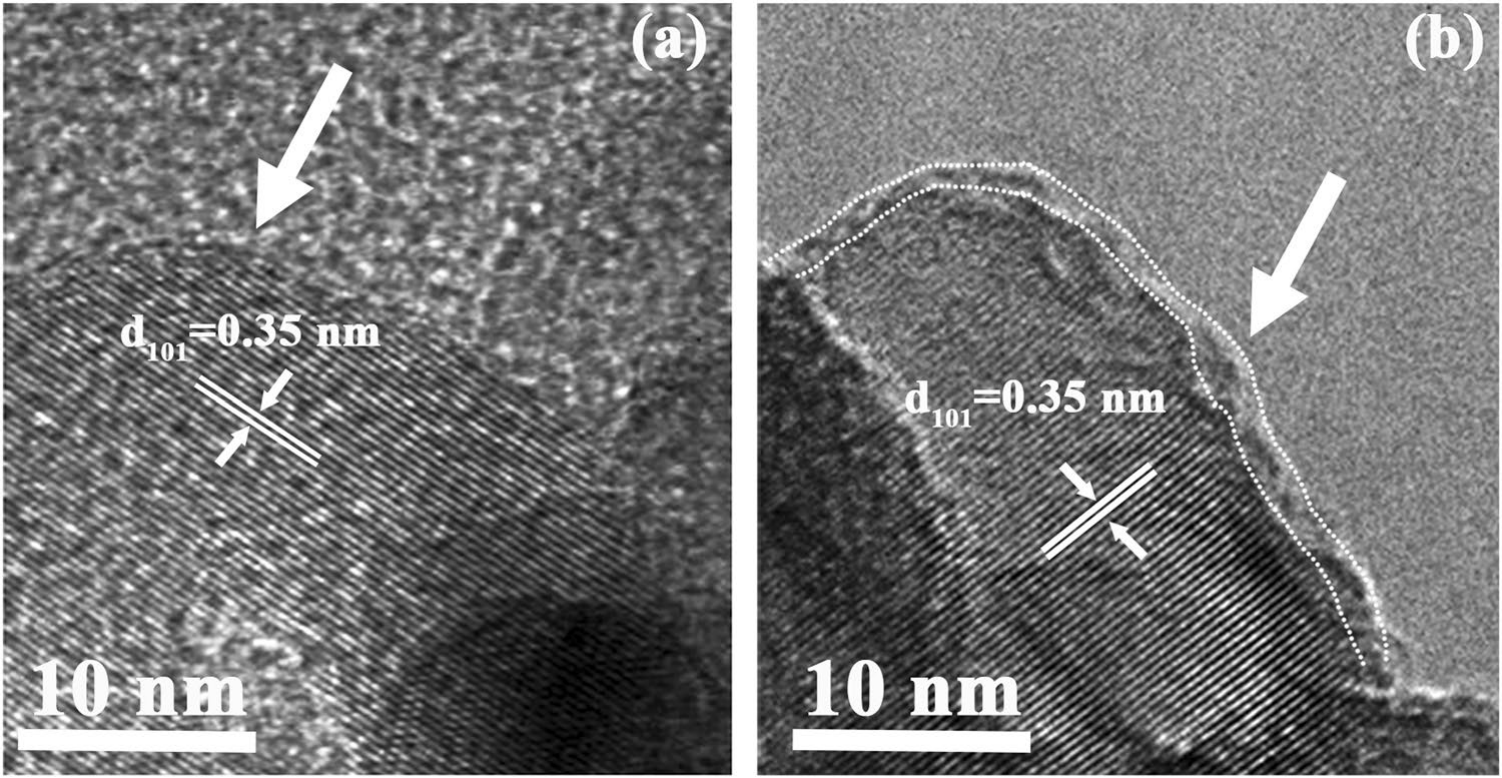
TEM images of (**a**) pristine TiO_2_ and (**b**) TiO_x_N_y_ coated TiO_2_ (TiO_2_@50TiN).

FESEM was performed to observe the morphology and crystal size of TiO_2_ and TiO_x_N_y_ coated TiO_2_ powders, as shown in Fig. S3. It can be found that the pristine TiO_2_ powders show well dispersed sphere of around 10–30 nm and aggregate together. After PEALD deposition, it can be seen that ultrathin TiO_x_N_y_ coating has no obvious effect on the morphology and crystal size of TiO_2_. All the samples exhibit the similar morphology. In order to thoroughly characterize the microstructure change of TiO_2_ after surface coating, high resolution transmission electron microscopy (HRTEM) was also applied to observe the microstructure of TiO_2_ and TiO_2_@50TiN. It can be found that pristine TiO_2_ exhibits good crystallinity with a sharp well-ordered surface (Fig. 3a). After 50 cycles TiN coating, there is an amorphous layer formed on the TiO_2_ surface of ~1 nm (Fig. 3b). It is supposed to be the ultrathin TiO_x_N_y_ coating formed after PEALD TiN deposition. Besides, both samples show a lattice spacing of 0.35 nm, which corresponds to the (101) planes of anatase TiO_2_. Therefore, it can be concluded from XPS spectra, Raman spectra, and HRTEM images that an amorphous ultrathin TiO_x_N_y_ was formed on the TiO_2_ surface.

**Figure 4:**
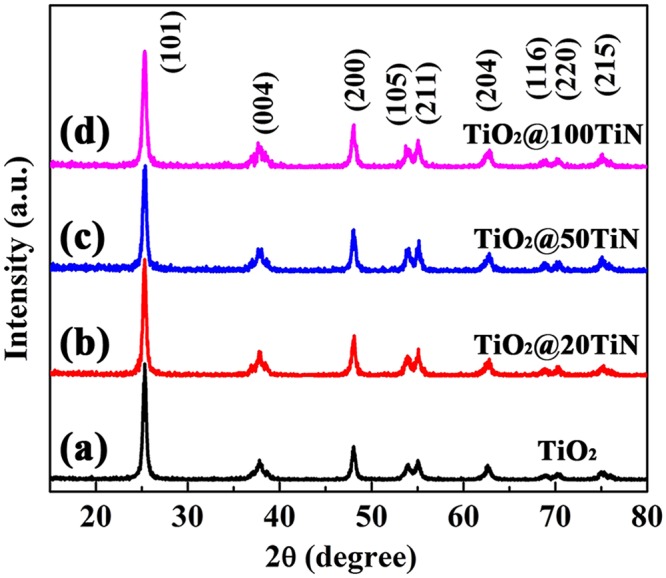
XRD patterns of (**a**) pristine TiO_2_, (**b**) TiO_2_@20TiN, (**c**) TiO_2_@50TiN, and (**d)** TiO_2_@100TiN.

The corresponding XRD patterns of pristine TiO_2_ and TiO_x_N_y_ coated TiO_2_ powders are shown in Fig. 4. All the samples exhibit the similar characteristic diffraction peaks at 25.4°, 37.9°, 48.0°, 54.1°, 63.0° etc., indicating good agreement with standard anatase TiO_2_ (JCPDS No. 71-1168). Besides, there are no other peaks such as Ti-N detected in the samples. In addition, the average crystal size and lattice parameters of different samples can be determined by XRD using Scherrer equation, as listed in Table 1. It can be found that the crystal size of all the samples is estimated to be around 19 nm, in agreement with SEM images. In addition, all the samples show nearly the same lattice parameters, indicating that ultrathin TiO_x_N_y_ surface coating does not change the crystal size and average unit cell dimension. Moreover, Nitrogen adsorption-desorption isotherms were also performed to measure the surface area of TiO_2_ powders, it can be found that all the samples exhibit nearly the same BET surface area of around 113 m^2^/g (Table 1 and Fig. S4).

**Table 1:** Parameters of pristine TiO_2_ and TiO_x_N_y_ coated TiO_2_ powders.

Sample	a_0_ (Å)	c_0_ (Å)	Crystallite size (nm)	BET surface area (m^2^/g)
TiO_2_	3.78	9.54	19.03	112.6
TiO_2_@20TiN	3.78	9.55	19.27	112.6
TiO_2_@50TiN	3.78	9.53	18.98	111.7
TiO_2_@100TiN	3.78	9.55	18.55	115.2

**Figure 5:**
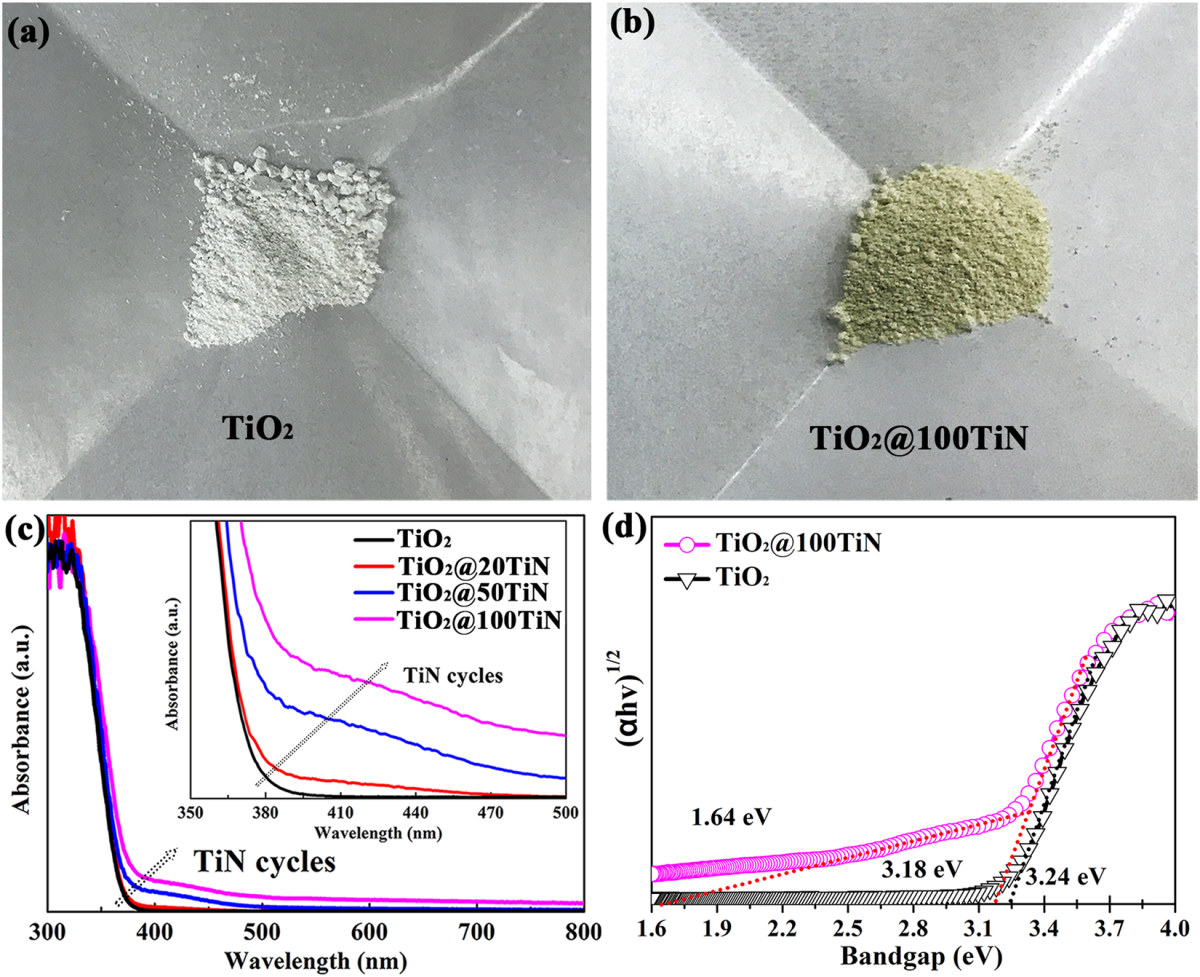
Optical photos of (**a**) pristine TiO_2_ and (**b**) TiO_2_@100TiN. (**c**) UV-Vis diffuse reflectance spectra of pristine TiO_2_ and TiO_x_N_y_ coated TiO_2_ powders. (**d**) The corresponding band gaps determination plots of pristine TiO_2_ and TiO_2_@100TiN.

Therefore, it can be concluded that ultrathin TiO_x_N_y_ coating can be formed on the surface of TiO_2_ powders. And this ultrathin surface coating doesn’t show obvious change in the morphology, crystal size, lattice parameters, and surface area of TiO_2_ nano powders. However, it can be clearly seen that there is a vivid color change of TiO_2_ powders from white to yellow after ultrathin TiO_x_N_y_ surface modification, as shown in Fig. 5a,b. Hence, UV-Vis diffuse reflectance spectra were conducted to explore the influence of ultrathin TiO_x_N_y_ surface coating on the visible light absorption of TiO_2_ powders, as shown in Fig. 5c. For comparison, the spectrum of pristine TiO_2_ powder is also illustrated. The absorption edge of pristine TiO_2_ is approximately 371 nm and does not show noticeable absorption in the visible region. However, all the TiO_x_N_y_ coated TiO_2_ samples exhibit distinct and meaningful absorption in the visible range from 390 to 500 nm, consistent with previous experimental results^20,44^. Furthermore, more TiO_x_N_y_ coating can induce more visible light absorption. For the indirect bandgap semiconductor, the relation between the absorption edge and the photon energy (hν) can be written as follows: (αhν)^1/2^ = A(hν - E_g_), where A is the absorption constant of the indirect band gap semiconductor material. The absorption coefficient (α) is determined from the scattering and reflectance spectra according to Kubelka-Munk theory. The indirect bandgap energies estimated from the intercept of the tangents to the plots are presented in Fig. 5d. The bandgap of pristine TiO_2_ powders is determined to be 3.24 eV. TiO_x_N_y_ coated TiO_2_ (100 cycles TiN) exhibits two bandgaps. The larger bandgap of 3.18 should be related to the TiO_2_ supporters. Besides, a smaller band gap of 1.64 eV can be assigned to the band gap value of TiO_x_N_y_ coating layer. Therefore, it can be concluded that ultrathin TiO_x_N_y_ surface modification layer with smaller band gap can facilitate the visible light absorption of TiO_2_ powders.

**Figure 6:**
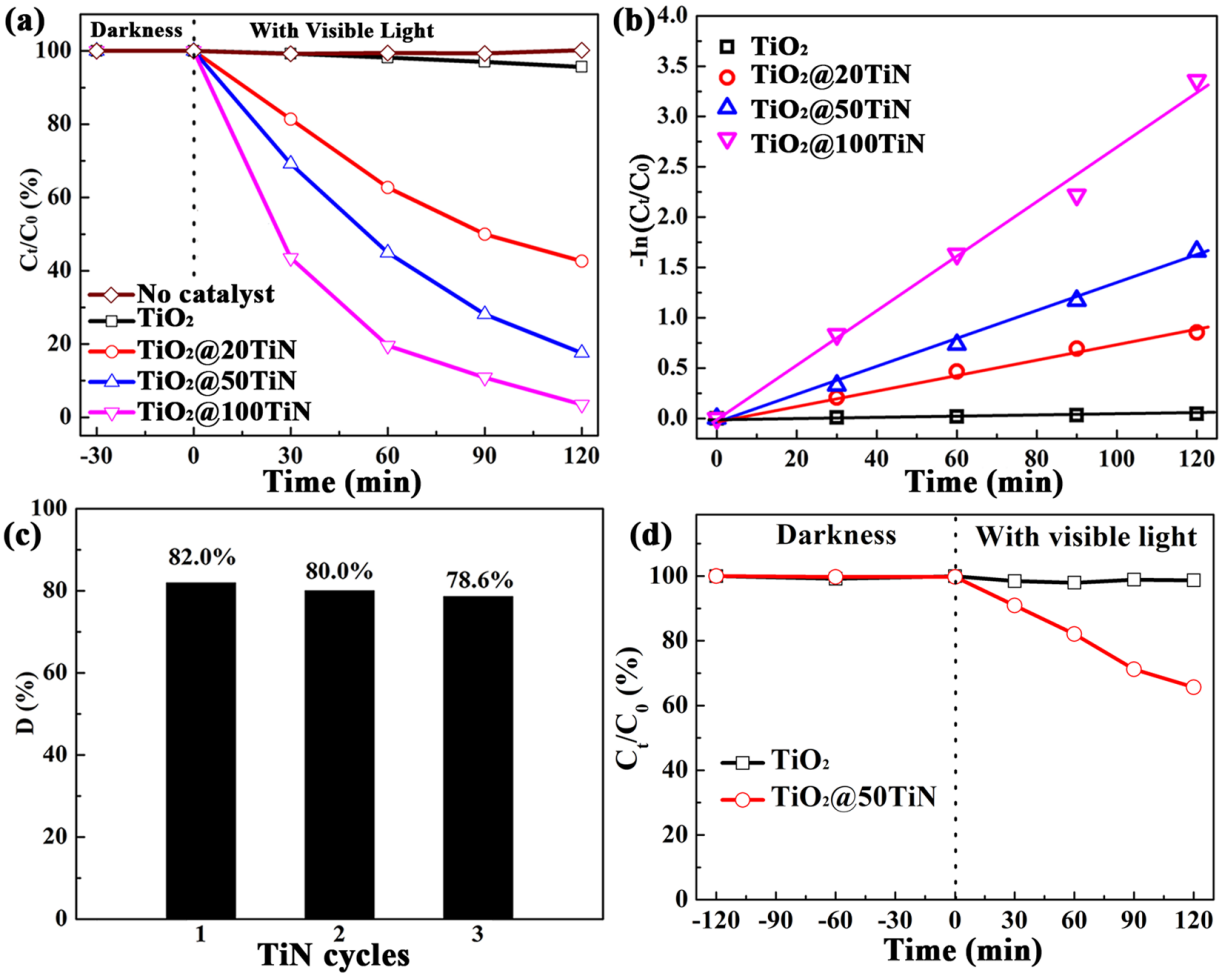
(**a**) Photocatalytic degradation of MO by using TiO_2_ and TiO_x_N_y_ coated TiO_2_ catalysts prepared by PEALD under visible-light irradiation, (**b**) the corresponding -ln(C_t_/C_0_) vs. time curves, (**c**) three cycles of MO degradation for TiO_2_@50TiN in 120 min, (**d**) photocatalytic degradation of phenol by using TiO_2_ and TiO_2_@50TiN catalysts under visible-light irradiation.

The photocatalytic activity of TiO_x_N_y_ coated TiO_2_ has been investigated carefully through degrading methyl orange (MO) under visible light irradiation, as shown in Fig. 6. All the samples exhibit negligible adsorption capacity of MO, as shown in Fig. S5. Meanwhile, almost no degradation of MO is observed in the absence of catalyst, indicating that MO is stable under visible light irradiation. As shown in Fig. 6a, pristine TiO_2_ shows very limited photocatalytic activity of ~4.4% in 120 min under visible light irradiation due to its large band gap. However, after ultrathin TiO_x_N_y_ coating with only 20 cycles PEALD TiN, a much-improved photocatalytic activity of ~57.3% is achieved. Moreover, the photocatalytic activity improves with increasing the TiN coating cycles, with the TiO_2_@100TiN exhibiting the highest degradation efficiency of ~96.5%. The experimental results were also fitted to the pseudo-first-order kinetics. At low initial pollutant concentration, the rate constant k was given by In(C_t_/C_0_) = −kt. Here, k and t represent the first-order rate constant (h^−1^), and the irradiation time (h), respectively. C_0_ is the initial concentration of MO, and C_t_ is the concentration at reaction time of t. The corresponding plots of -In(C_t_/C_0_) versus the irradiation time for photodegradation of MO are shown in Fig. 6b. A linear relation between -In(C_t_/C_0_) and the irradiation time has verified that the photodegradation of MO using TiO_x_N_y_ coated TiO_2_ catalyst follows the first-order kinetics. TiO_x_N_y_ coated TiO_2_ exhibit the kinetic constants of 1.62 h^−1^ (TiO_2_@100 TiN), 0.82 h^−1^ (TiO_2_@50 TiN), and 0.45 h^−1^ (TiO_2_@20 TiN), which are much larger than pristine TiO_2_ of 0.023 h^−1^. Apparently, ultrathin TiO_x_N_y_ coating can greatly improve the visible light photodegradation efficiency of MO due to its absorption in visible light spectrum. In order to evaluate the stability of the photocatalyst, the recycling experiments about MO photodegradation were performed with TiO_2_@50TiN catalyst. As shown in Fig. 6c, the photocatalytic activity of TiO_2_@50TiN exhibits an extremely limited decline for three times. The degradation efficiency of MO solution is nearly the same for three recycling experiment under 120 min irradiation, exhibiting wonderful recycling ability.

Moreover, colorless phenol was also adopted to evaluate the visible photocatalytic performance of TiO_2_@50TiN. As shown in Fig. 6d, it can be seen that both pure TiO_2_ and TiO_2_@50TiN exhibit negligible absorption for phenol molecule in the darkness. Pure TiO_2_ powder shows no photocatalytic activity towards degrading phenol molecule. There is hardly any degradation of phenol for TiO_2_ with 2 h visible irradiation. However, after modification with 50 cycles of TiN, the TiO_2_@50TiN powders exhibit visible photocatalytic activity for phenol, around 34.3% of phenol can degrade in 2 h. Therefore, it can also be demonstrated that surface modification with PEALD TiN can greatly improve the visible photocatalytic activity of TiO_2_.

**Figure 7:**
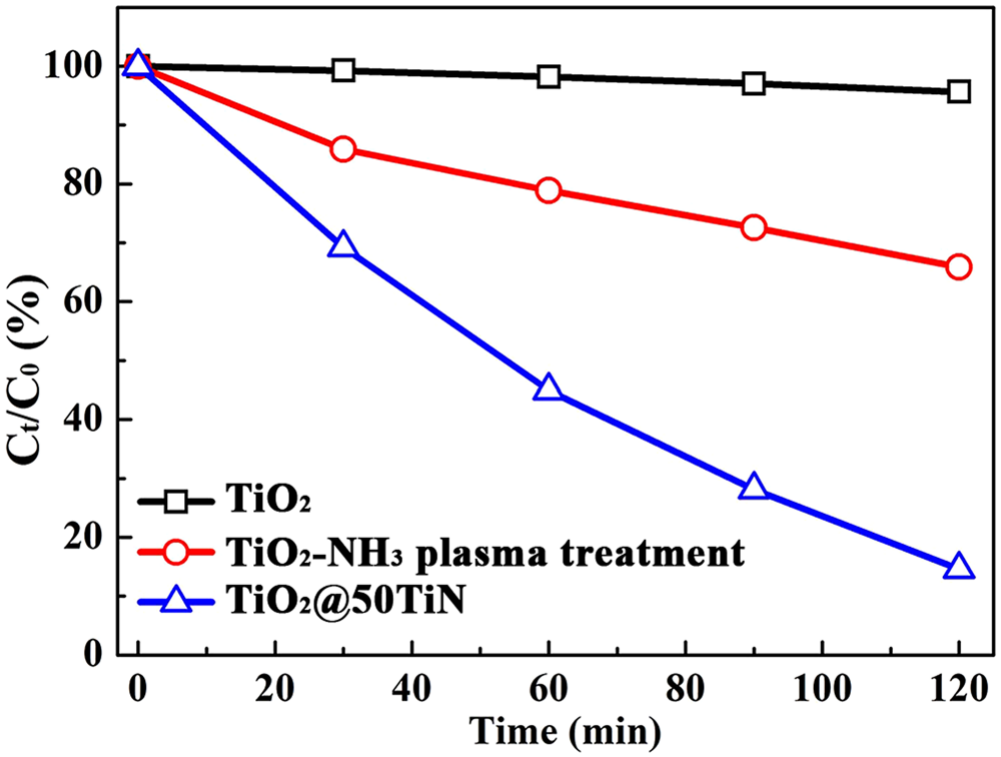
Photocatalytic degradation of MO by using NH_3_ plasma treated TiO_2_ and TiO_x_N_y_ coated TiO_2_ catalysts under visible-light irradiation.

As reported previously, visible light active photocatalytic N-doped TiO_2_ can be achieved by annealing TiO_2_ under NH_3_ flux at high temperature^17^. Thus, a control experiment using TiO_2_ photocatalyst treated by NH_3_ plasma at 360 °C was also performed, as shown in Fig. 7. It can be seen that NH_3_ plasma treatment can only slightly improve the photocatalytic activity of TiO_2_, the photocatalytic activity is much lower than the TiO_x_N_y_ coated sample. It can be concluded that, in order to achieve highly visible light active N-TiO_2_ based photocatalyst, it is easier and more effective to coat TiO_x_N_y_ thin film on TiO_2_ than replacing O with N under NH_3_ flux at high temperature.

**Figure 8:**
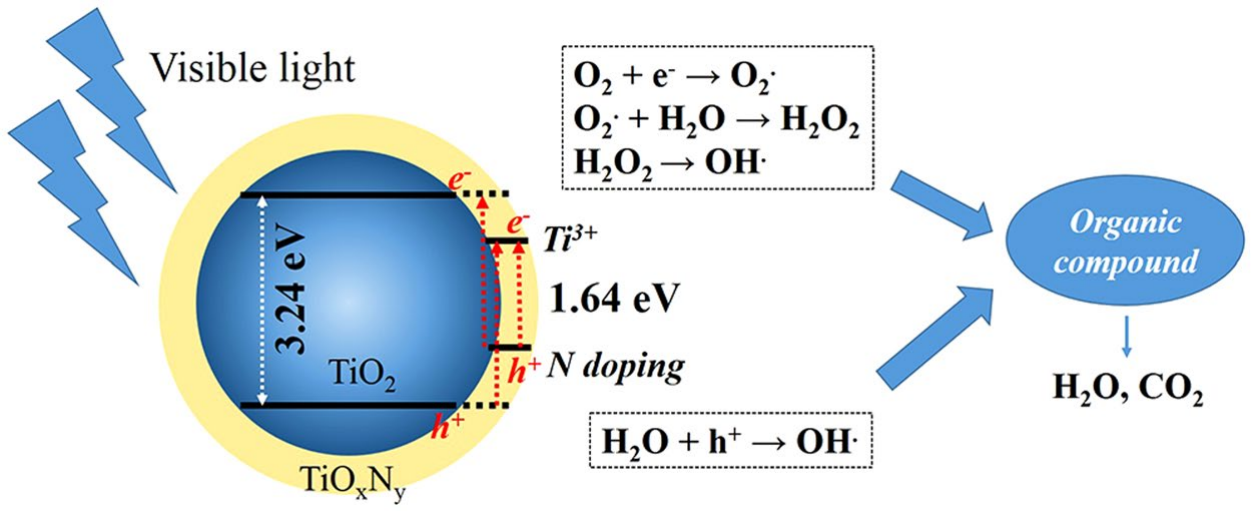
Proposed mechanism of TiO_x_N_y_ coated TiO_2_ for the degradation of MO under visible light irradiation.

The photocatalytic mechanism of TiO_x_N_y_ coated TiO_2_ is also proposed. There are a large number of reports focusing on the photocatalytic activity mechanism of N-doped TiO_2_. It has been demonstrated that both N doping and Ti^3+^ can contribute to narrowing the band gap of TiO_2_^21^, the band gap alignment and charge transfer of TiO_2_@TiO_x_N_y_ is shown in Fig. 8. It is widely accepted that N doping can form a new substitution N 2p band above the O 2p valance band. While the Ti^3+^ sites exhibit the 3d orbital in the band gap, which is found to below the bottom of the conduction band^21^. Therefore, TiO_x_N_y_ coated TiO_2_ exhibits a small band gap value of ~1.64 eV here, which can absorb the visible light. Upon visible light irradiation, electrons can transfer into the conduction band of TiO_2_ and Ti^3+^ sites, reducing O_2_ to form O_2_. radicals. The holes (h^+^) formed in the valance band and N doping sites would react with H_2_O to produce OH. radicals. Both radicals are responsible for the degradation of MO under visible light, as shown in Fig. 8. It should be noted that only an ultrathin TiO_x_N_y_ coating here can significantly improve the visible light photocatalytic activity of commercial TiO_2_ powders. Therefore, maybe it’s needless to synthesize the monolithic N-doped TiO_2_ composites, adopting ultrathin TiO_x_N_y_ coating can be an effective approach to prepare visible light active photocatalysts. In addition, surface coating or modification using ALD technology can be easily extended to other supporters, such as porous materials, nanowires, and so on.

## Conclusions

In summary, a novel and facile approach to prepare ultrathin TiO_x_N_y_ coated TiO_2_ composite by PEALD has been developed to promote the application of TiO_2_ photocatalyst under visible light. An ultrathin TiO_x_N_y_ film can be formed perfectly on the surface of TiO_2_ powders using PEALD. Introducing ultrathin TiO_x_N_y_ coating with smaller bandgap of ~1.64 eV can facilitate the absorption of TiO_2_ in visible light spectrum. As a result, this ultrathin TiO_x_N_y_ coating can extraordinarily improve the photocatalytic activity of commercials TiO_2_ powders towards degrading both MO and phenol under visible light. TiO_2_@100TiN prepared by PEALD photocatalyst could nearly degrade MO completely (~96.5%) in 120 min under visible light irradiation, while pristine TiO_2_ shows very weak photoactivity of only 4.4%. Moreover, TiO_x_N_y_ coated TiO_2_ photocatalyst is quite stable and reusable. Therefore, this surface modification using PEALD is an extremely promising route that could also be extended to other supporters to prepare visible light active photocatalysts. These results presented in this work could open a new window to the future design and synthesis of visible light photocatalysts.

## Methods

### Chemicals

In ALD process, Titanium tetrachloride (TiCl_4_) (5N, Suzhou Fornano Corporation Ltd.) and NH_3_ plasma were used as Ti precursor and Nitrogen sources, respectively. High pure N_2_ (5N) and Ar (5N) were used as carrier and purge gas. Commercial anatase TiO_2_ powders (Nanjing Haitai nano materials Co) with diameter of ~20 nm were used as supporters. Methyl Orange (MO, C_14_H_14_N_3_NaO_3_S, J&K Scientific) and phenol was prepared into 4 mg L^−1^ with Milli-Q water.

### Preparation of TiO_x_N_y_ modified TiO_2_ powder

TiO_2_ powders were loaded into a special powder container with porous mesh. The schematic diagram of coating TiO_2_ powders by PEALD TiN is shown in Fig. S6. TiCl_4_ and NH_3_ plasma were used as precursors for TiN deposition. Plasma power and NH_3_ gas flow rate were 2500 W and 160 sccm, respectively. And it is a remote plasma source. Pure N_2_ (5N) and Ar (5N) were used as carrier/purge gas for TiCl_4_ and NH_3_ plasma, respectively. Various cycles of TiN were deposited onto TiO_2_ surface at 360 °C, where one cycle consisted of 2 s TiCl_4_ injection, 10 s purging, 24 s NH_3_ plasma injection, and 6 s purging. Long dosing/purging time was applied to gain conformal coating on nano powders. In this work, the samples coated by 20, 50, 100 cycles of TiN are termed as TiO_2_@20TiN, TiO_2_@50TiN and TiO_2_@100TiN, respectively. As a control experiment, TiO_2_ powders were treated by NH_3_ plasma at 360 °C for 20 min, which is equal to the NH_3_ plasma injection time of 50 cycles of PEALD TiN.

### Characterization

The chemical feature was investigated by X-ray photoelectron spectroscopy (XPS, Thermo Fisher K-Alpha) with standard Al Kα (1486.7 eV) X-ray source. The binding energies were calibrated with respect to the signal from the adventitious carbon (binding energy = 284.6 eV). Raman spectra of TiO_2_ were collected by a confocal Raman microscope (LabRAM HR Evolution, Horiba) with excitation laser wavelength of 632.8 nm. An objective lens is employed to focus the excitation laser on the substrate and collect the Raman signal. The microstructure and morphology were examined by filed effect scanning electron microscopy (FESEM, Ultra55, ZEISS) and high-resolution transmission electron microscopy (HRTEM, Tecnai F20 S-Twin, FEI). Crystallinity and phase structures of powders were analyzed by a Rigaku-D/MAX 2000X-ray diffraction (XRD) system with Cu Kα radiation. The Brunauer-Emmett-Teller (BET) surface area was estimated by a surface area apparatus (TriStar-3000, Micromeritics). UV-visible absorption spectra were recorded by a UV-vis-NIR spectrophotometer (UV-3600, Shimadzu).

### Photocatalytic activity

The photocatalytic activity of as-prepared photocatalysts was evaluated via the degradation of methyl orange (MO) or phenol in aqueous solution. A solar simulator (300 W Xe lamp, MircoSolar300, PerfectLight) with a 420 nm cut-off filter provides the visible-light irradiation. The lamp was located at 15 cm away from the reaction solution. 100 mg catalyst and 100 ml of aqueous solution containing 4 mg L^−1^ MO or phenol were placed in a glass reactor with continuous stirring at 500 rpm. Prior to irradiation, the pollutant solutions suspended with photocatalysts were stirred in absence of light for 30 min (MO) or 2 h (phenol) to attain the equilibrium adsorption/desorption between photocatalyst powders and organic molecules. During the reaction, the temperature was maintained at 25 ± 1 °C using cooling water. For each given irradiation time, about 3 mL of the reacted solution was withdrawn and centrifuged at 10,000 rpm for 10 min to remove the photocatalyst. Then, the concentration of the centrifuged solution was determined by a UV-vis-NIR spectrophotometer, measuring the maximum absorption of MO at 464 nm and phenol at 270 nm.

### Stability test of photocatalysts

In order to evaluate the stability of the photocatalysts, a recycled usage experiment was carried out. 100 mg TiO_2_@50TiN photocatalyst was suspended in a 100 mL of 4 mg L^−1^ solution of MO and irradiated under Xe lamp for 120 min. The photocatalysts were collected and washed by distilled water and ethanol, then dried in the oven at 100 °C for 12 h. Finally, the photocatalyst was reused again for the second cycle of degradation with a fresh dye solution. This process was about to repeat up to 3 times of application.

## Electronic supplementary material

Supplementary info

## References

[1] Fujishima A (1972). Electrochemical photolysis of water at a semiconductor electrode. Nature.

[2] Chen X, Mao SS (2007). Titanium dioxide nanomaterials: synthesis, properties, modifications, and applications. Chem. Rev.

[3] Pelaez M (2012). A review on the visible light active titanium dioxide photocatalysts for environmental applications. Appl. Catal. B Environ..

[4] Linsebigler AL, Lu G, Yates JT (1995). Photocatalysis on TiO_2_ surfaces: principles, mechanisms, and selected results. Chem. Rev.

[5] Ohno T, Mitsui T, Matsumura M (2003). Photocatalytic activity of S-doped TiO_2_ photocatalyst under visible light. Chem. Lett.

[6] Diwald O (2004). Photochemical activity of nitrogen-doped rutile TiO_2_ (110) in visible light. J. Phys. Chem. B.

[7] Yan X (2017). The interplay of sulfur doping and surface hydroxyl in band gap engineering: Mesoporous sulfur-doped TiO_2_ coupled with magnetite as a recyclable, efficient, visible light active photocatalyst for water purification. Appl. Catal. B Environ.

[8] Zhang Z, Zhang L, Hedhili MN, Zhang H, Wang P (2012). Plasmonic gold nanocrystals coupled with photonic crystal seamlessly on TiO_2_ nanotube photoelectrodes for efficient visible light photoelectrochemical water splitting. Nano Lett.

[9] Li G-S, Zhang D-Q, Yu JC (2009). A new visible-light photocatalyst: CdS quantum dots embedded mesoporous TiO_2_. Environ. Sci. Technol.

[10] Choi T, Kim J-S, Kim JH (2016). Transparent nitrogen doped TiO_2_/WO_3_ composite films for self-cleaning glass applications with improved photodegradation activity. Adv. Powder Technol.

[11] Guo X (2017). Porous TiB_2_-TiC/TiO_2_ heterostructures: Synthesis and enhanced photocatalytic properties from nanosheets to sweetened rolls. Appl. Catal. B Environ.

[12] Fujishima A, Zhang X, Tryk DA (2008). TiO_2_ photocatalysis and related surface phenomena. Surf. Sci. Rep.

[13] Zhang J, Wu Y, Xing M, Leghari SAK, Sajjad S (2010). Development of modified N doped TiO_2_ photocatalyst with metals, nonmetals and metal oxides. Energ. Environ. Sci.

[14] Burda C (2003). Enhanced nitrogen doping in TiO_2_ nanoparticles. Nano Lett.

[15] Chen X, Lou YB, Samia AC, Burda C, Gole JL (2005). Formation of Oxynitride as the Photocatalytic Enhancing Site in Nitrogen-Doped Titania Nanocatalysts: Comparison to a Commercial Nanopowder. Adv. Funct. Mater.

[16] Shifu C, Lei C, Shen G, Gengyu C (2005). The preparation of nitrogen-doped photocatalyst TiO_2−x_N_x_ by ball milling. Chem. Phys. Lett.

[17] Lai Y-K (2010). Nitrogen-doped TiO_2_ nanotube array films with enhanced photocatalytic activity under various light sources. J. Hazard. Mater.

[18] Martínez-Ferrero E (2007). Nanostructured Titanium Oxynitride Porous Thin Films as Efficient Visible-Active Photocatalysts. Adv. Funct. Mater.

[19] Quesada-Cabrera R (2017). On the apparent visible-light and enhanced UV-light photocatalytic activity of nitrogen-doped TiO_2_ thin films. J. Photochem. Photobio. A Chem.

[20] Lee A (2017). Conformal Nitrogen-Doped TiO_2_ Photocatalytic Coatings for Sunlight-Activated Membranes. Adv. Sustainable Sys..

[21] Yang G, Jiang Z, Shi H, Xiao T, Yan Z (2010). Preparation of highly visible-light active N-doped TiO_2_ photocatalyst. J. Mater. Chem.

[22] Luong NS (2017). Highly Visible Light Activity of Nitrogen Doped TiO_2_ Prepared by Sol-Gel Approach. J. Electron. Mater.

[23] Liu M, Li X, Karuturi SK, Tok AIY, Fan HJ (2012). Atomic layer deposition for nanofabrication and interface engineering. Nanoscale.

[24] Knez M, Nielsch K, Niinistö L (2007). Synthesis and surface engineering of complex nanostructures by atomic layer deposition. Adv. Mater.

[25] Meng X (2017). Atomic Layer Deposition for Nanomaterials Synthesis and Functionalization in EnergyTechnology. Mater. Horiz..

[26] Cao Y-Q (2017). Atomic-Layer-Deposition Assisted Formation of Wafer-Scale Double-Layer Metal Nanoparticles with Tunable Nanogap for Surface-Enhanced Raman Scattering. Sci. Rep.

[27] Meng X, Yang X-Q, Sun X (2012). Emerging Applications of Atomic Layer Deposition for Lithium-Ion Battery Studies. Adv. Mater.

[28] Cao Y, Meng X, Elam JW (2016). Atomic Layer Deposition of Li_x_Al_y_S Solid-State Electrolytes for Stabilizing Lithium-Metal Anodes. Chem Electro Chem.

[29] Guan C, Wang J (2016). Recent Development of Advanced Electrode Materials by Atomic Layer Deposition for Electrochemical Energy Storage. Adv. Sci.

[30] Ahmed B, Xia C, Alshareef HN (2016). Electrode surface engineering by atomic layer deposition: A promising pathway toward better energy storage. Nano Today.

[31] Cao Y-Q (2017). ZnO/ZnS Core-Shell Nanowires Arrays on Ni Foam Prepared by Atomic Layer Deposition for High Performance Supercapacitors. J. Electrochem. Soc.

[32] Lu J, Elam JW, Stair PC (2016). Atomic layer deposition-Sequential self-limiting surface reactions for advanced catalyst “bottom-up” synthesis. Surf. Sci. Rep.

[33] Cao YQ (2015). Photocatalytic activity and photocorrosion of atomic layer deposited ZnO ultrathin films for the degradation of methylene blue. Nanotechnology.

[34] Wang T, Luo Z, Li C, Gong J (2014). Controllable fabrication of nanostructured materials for photoelectrochemical water splitting via atomic layer deposition. Chem. Soc. Rev.

[35] Profijt H, Potts S, Van de Sanden M, Kessels W (2011). Plasma-assisted atomic layer deposition: Basics, opportunities, and challenges. J. Vac. Sci. Technol. A.

[36] Van Bui H (2011). Growth kinetics and oxidation mechanism of ALD TiN thin films monitored by *in situ* spectroscopic ellipsometry. J. Electrochem. Soc.

[37] Saha NC, Tompkins HG (1992). Titanium nitride oxidation chemistry: An X-ray photoelectron spectroscopy study. J. Appl. Phys.

[38] Asahi R, Morikawa T, Ohwaki T, Aoki K, Taga Y (2001). Visible-light photocatalysis in nitrogen-doped titanium oxides. Science.

[39] Shultz AN (1995). Comparative second harmonic generation and X-ray photoelectron spectroscopy studies of the UV creation and O_2_ healing of Ti^3+^ defects on (110) rutile TiO_2_ surfaces. Surf. Sci.

[40] Chen Y (2016). Microwave-assisted ionic liquid synthesis of Ti^3+^ self-doped TiO_2_ hollow nanocrystals with enhanced visible-light photoactivity. Appl. Catal. B Environ.

[41] Livraghi S (2006). Origin of photoactivity of nitrogen-doped titanium dioxide under visible light. J. Am. Chem. Soc.

[42] Yanagisawa K, Ovenstone J (1999). Crystallization of anatase from amorphous titania using the hydrothermal technique: effects of starting material and temperature. J. Phys. Chem. B.

[43] Wang J, Zhu W, Zhang Y, Liu S (2007). An efficient two-step technique for nitrogen-doped titanium dioxide synthesizing: visible-light-induced photodecomposition of methylene blue. J. Phys. Chem. C.

[44] Irie H, Watanabe Y, Hashimoto K (2003). Nitrogen-concentration dependence on photocatalytic activity of TiO_2−x_N_x_ powders. J. Phys. Chem. B.

