# Supplementary material for: TiOxNy Modified TiO2 Powders Prepared by Plasma Enhanced Atomic Layer Deposition for Highly Visible Light Photocatalysis
Source: Sci Rep. 2018 Aug 14;8:12131. doi: 10.1038/s41598-018-30726-w (PMC6092356; doi:10.1038/s41598-018-30726-w)
Supplement: Supplementary file 1 — Supplementary info [file 41598_2018_30726_MOESM1_ESM.docx]

Supplementary information

**TiO_x_N_y_ Modified TiO_2_ Powders Prepared by Plasma Enhanced Atomic Layer Deposition for Highly Visible Light Photocatalysis**

**Yan-Qiang Cao, Xi-Rui Zhao, Jun Chen, Wei Zhang, Min Li, Lin Zhu, Xue-Jin Zhang, Di Wu and Ai-Dong Li***

National Laboratory of Solid State Microstructures and Department of Materials Science and Engineering, College of Engineering and Applied Sciences, Collaborative Innovation Center of Advanced Microstructures, Nanjing University, Nanjing 210093, People’s Republic of China.

Correspondence and requests for materials should be addressed to A.D. L. (adli@nju.edu.cn)


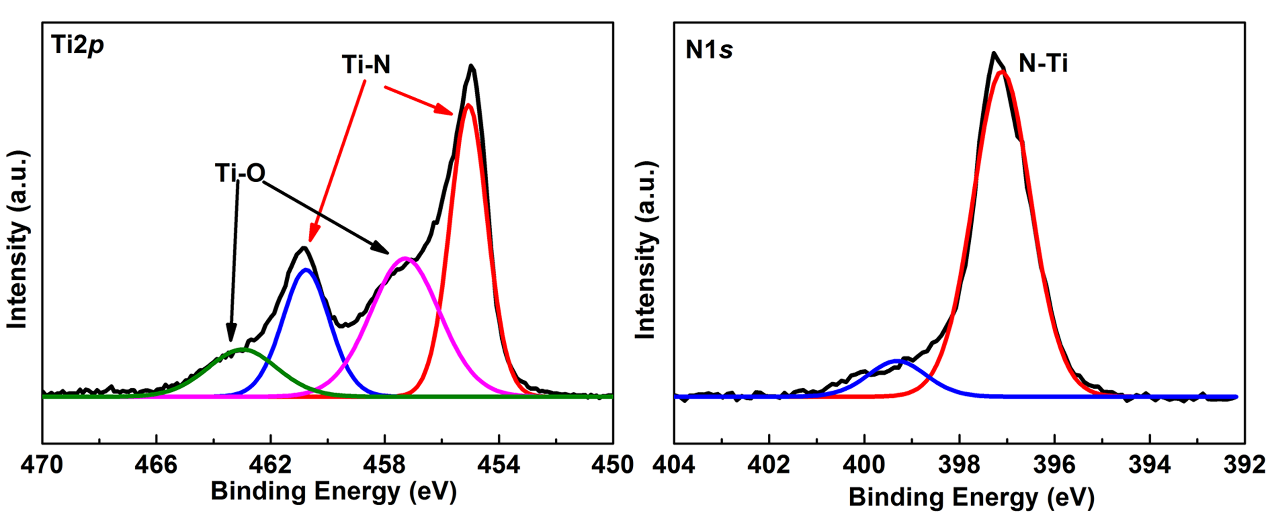


**Figure S1.** (a) Ti 2p and (b) N 1s XPS spectra of TiN film deposited by PEALD on Si.


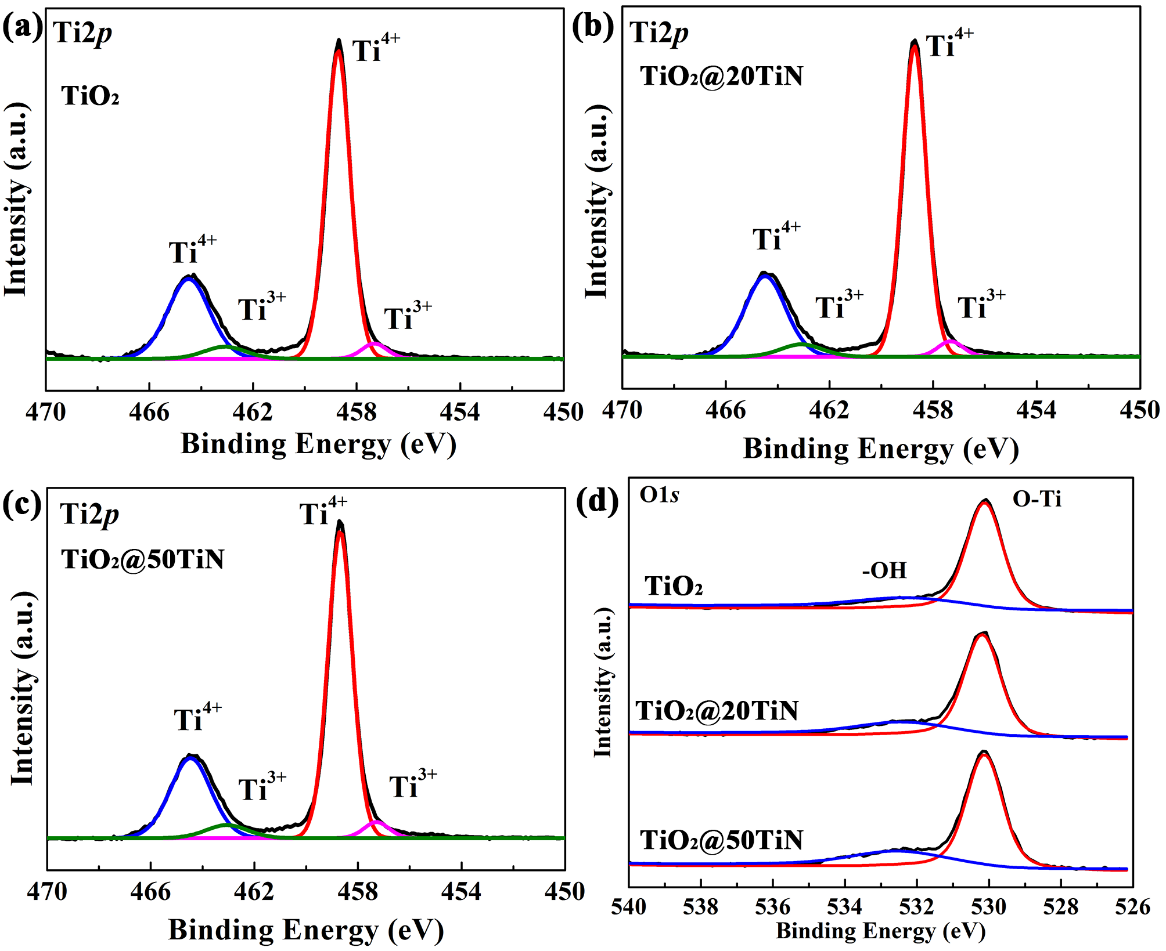


**Figure S2.** Ti 2p XPS spectra of (a) pristine TiO_2_, (b) TiO_2_@20TiN and (c) TiO_2_@50TiN, (d) O 1s XPS spectra of pristine TiO_2_ and TiO_x_N_y_ coated TiO_2_ by PEALD.


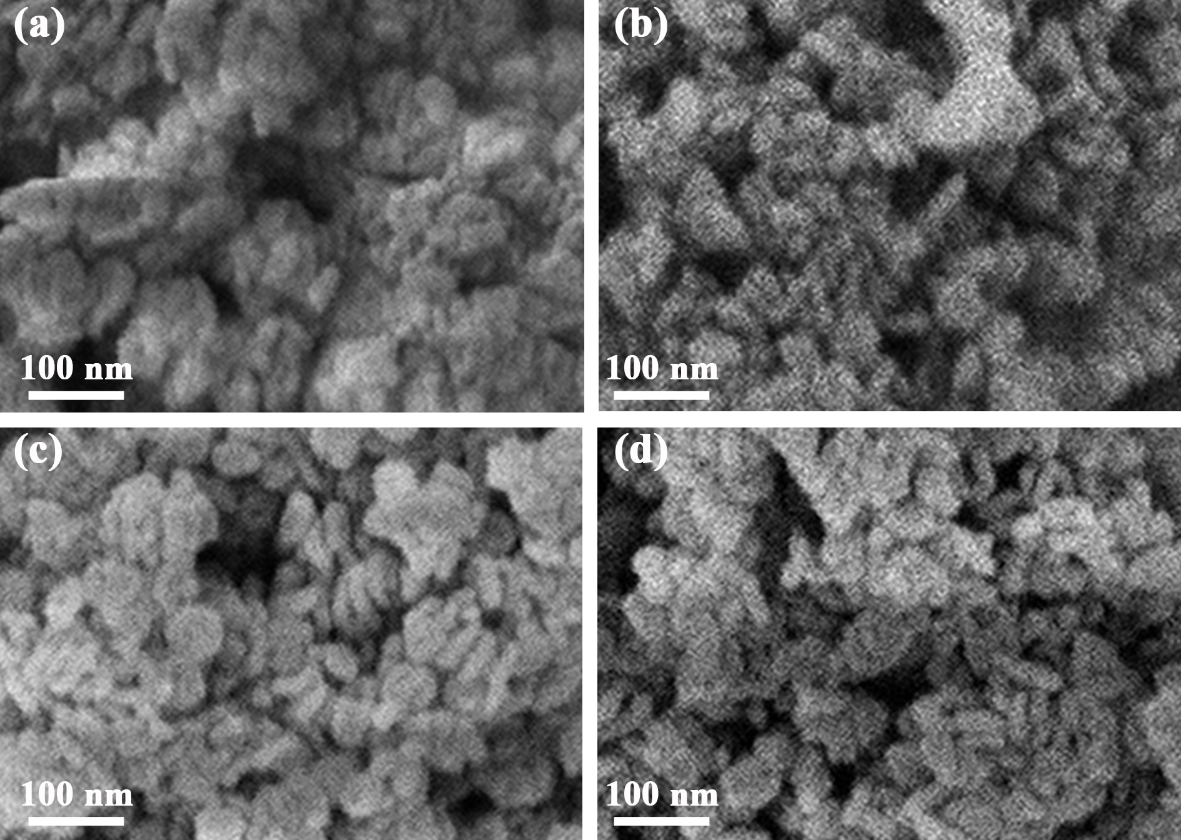


**Figure S3.** SEM images of (a) pristine TiO_2_, (b) TiO_2_@20TiN, (c) TiO_2_@50TiN, and (d) TiO_2_@100TiN.


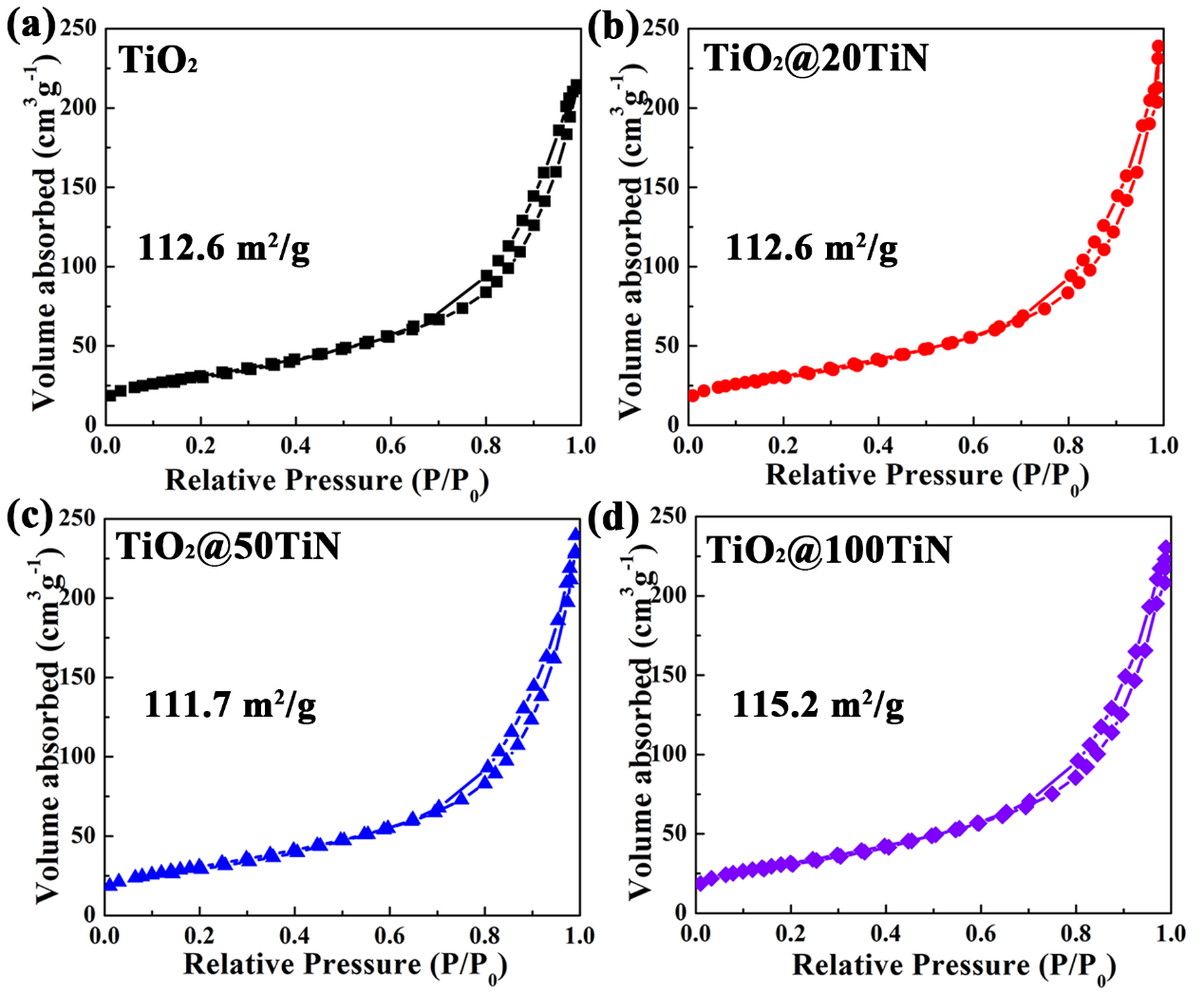


**Figure S4.** Nitrogen adsorption-desorption isotherms of (a) pristine TiO_2_, (b) TiO_2_@20TiN, (c) TiO_2_@50TiN, and (d) TiO_2_@100TiN by PEALD.


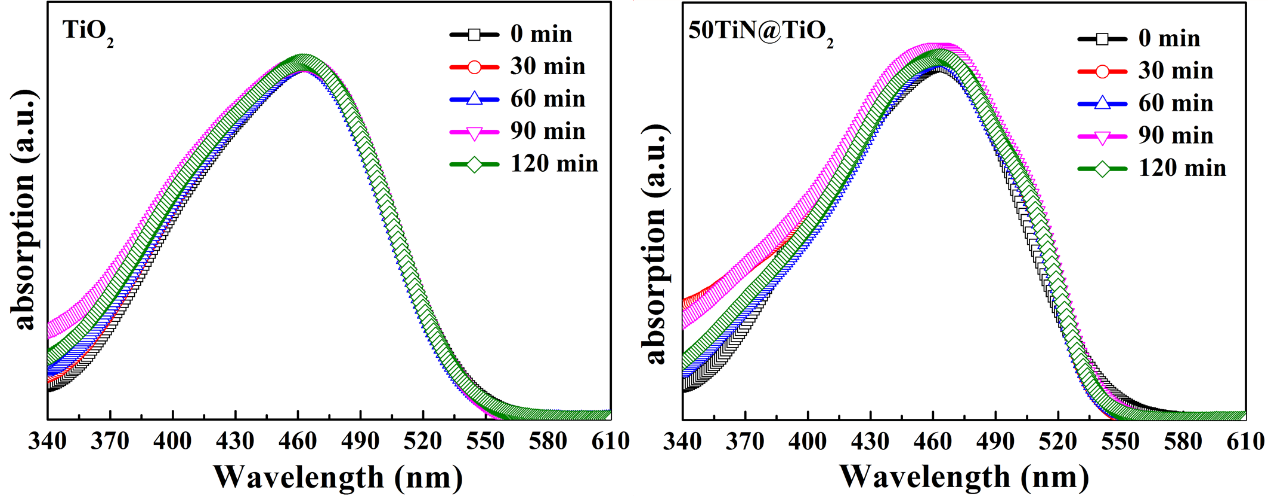


**Figure S5.** The characteristic absorption of MO with (a) TiO_2_ and 50TiN@TiO_2_ catalyst in the darkness.


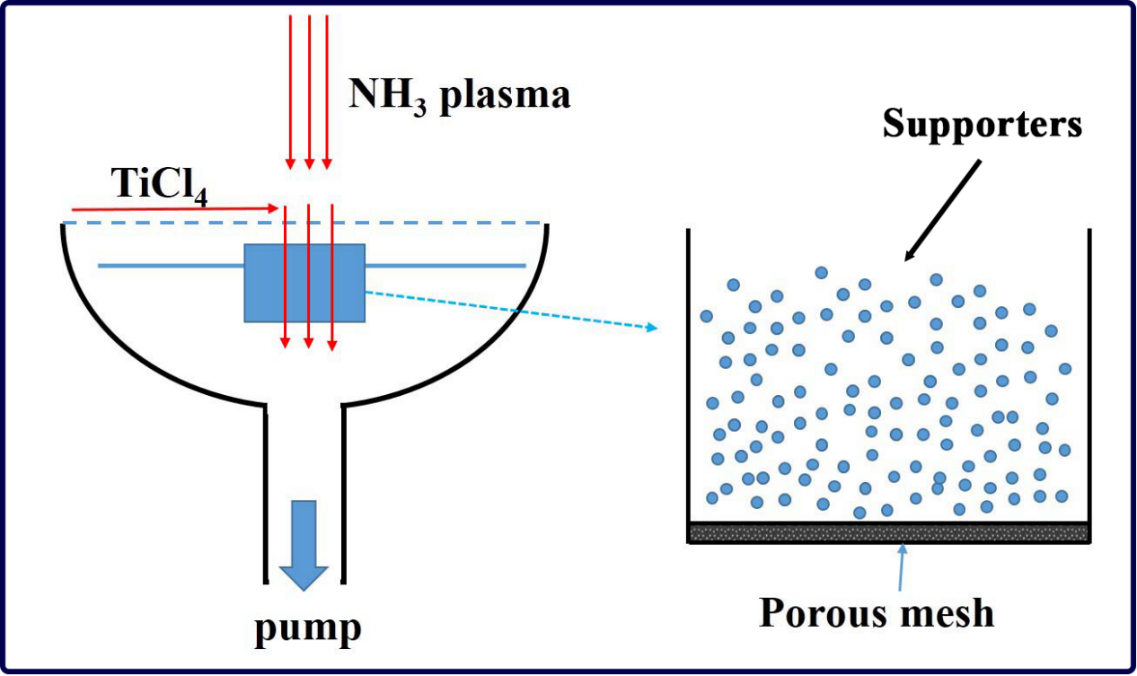


**Figure S6.** The schematic diagram of coating TiO_2_ powders by PEALD TiN.


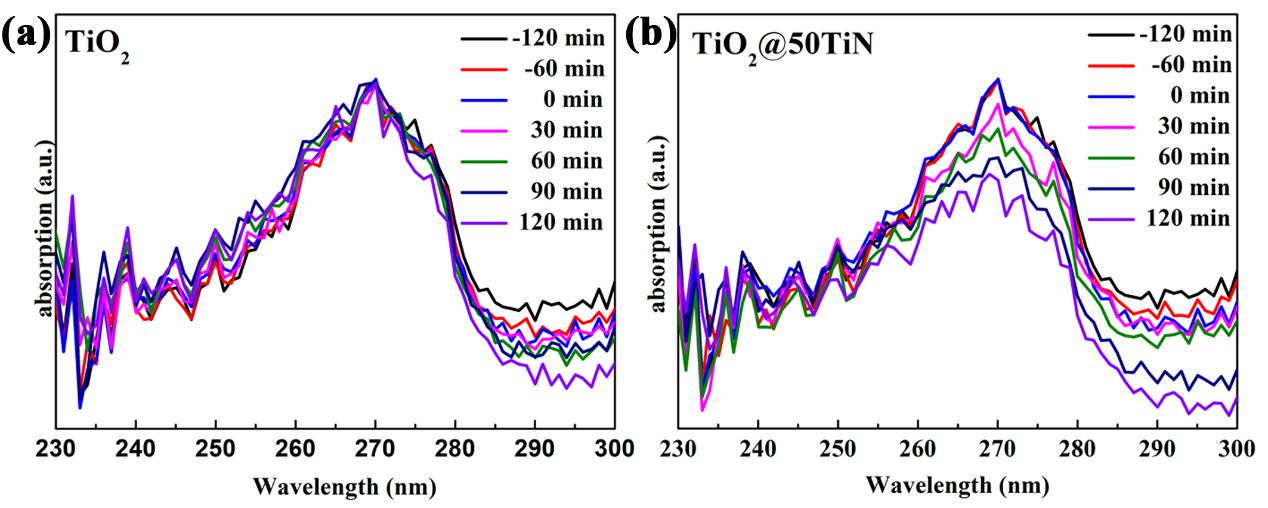


**Figure S7.** The characteristic absorption of phenol with (a) TiO_2_ and 50TiN@TiO_2_ catalyst under visible light irradiation.
